# Hierarchically electrospraying a PLGA@chitosan sphere-in-sphere composite microsphere for multi-drug-controlled release

**DOI:** 10.1093/rb/rbaa009

**Published:** 2020-04-14

**Authors:** Zhu Liu, Weilong Ye, Jingchuan Zheng, Qindong Wang, Guowu Ma, Huiying Liu, Xiumei Wang

**Affiliations:** r1 State Key Laboratory of New Ceramic & Fine Processing, School of Materials Science and Engineering, Tsinghua University, No. 1 Qinghuayuan, Beijing 100084, China; r2 Department of Prosthodontics, School of Stomatology, Dalian Medical University, No.9 west section, Lvshunnan Road, Dalian 116044, China

**Keywords:** PLGA, chitosan, electrospray, composite microsphere

## Abstract

Sequential administration and controlled release of different drugs are of vital importance for regulating cellular behaviors and tissue regeneration, which usually demands appropriate carriers like microspheres (MS) to control drugs releases. Electrospray has been proven an effective technique to prepare MS with uniform particle size and high drug-loading rate. In this study, we applied electrospray to simply and hierarchically fabricate sphere-in-sphere composite microspheres, with smaller poly(lactic-co-glycolic acid) MS (∼8–10 μm in diameter) embedded in a larger chitosan MS (∼250–300 μm in diameter). The scanning electron microscopy images revealed highly uniform MS that can be accurately controlled by adjusting the nozzle diameter or voltage. Two kinds of model drugs, bovine serum albumin and chlorhexidine acetate, were encapsulated in the microspheres. The fluorescence-labeled rhodamine-fluoresceine isothiocyanate (Rho-FITC) and ultraviolet (UV) spectrophotometry results suggested that loaded drugs got excellent distribution in microspheres, as well as sustained, slow release *in vitro*. In addition, far-UV circular dichroism and matrix-assisted laser desorption/ionization time-of-flight mass spectrometry (MALDI-TOF-MS) results indicated original secondary structure and molecular weight of drugs after electrospraying. Generally speaking, our research proposed a modified hierarchically electrospraying technique to prepare sphere-in-sphere composite MS with two different drugs loaded, which could be applied in sequential, multi-modality therapy.

## Introduction

Programmed sequential delivery of multiple drugs is currently considered an important issue for regulating cellular behaviors and tissue regeneration by targeting the demands for different dose of drugs at distinct healing phases. Therefore, developing appropriate drug delivery systems with tunable sequential drug release ability and good biocompatibility is of vital importance to obtain more effective therapeutic effects. Microspheres (MS) and microcapsules are widely studied in tissue engineering and pharmaceutics, which can encapsulate and protect various drugs or cytokines to enable long-term and sustained release, so that these drugs can exert functions on cells and tissues in a controlled manner [1–3]. 

Poly(lactic-co-glycolic acid) (PLGA) is a representative biodegradable polymer material with good biocompatibility and stability. Multiple medical devices made of PLGA have already been approved by the US FDA and commonly used as scaffolds for tissue engineering or carriers for various small molecule drugs, proteins and genes [4–6]. With the PLGA MS degrading gradually, the loaded drugs get released and exert bioactivities *in vitro* or *in vivo*. But the degraded products of PLGA, lactic acid and glycolic acid, provide an acidic environment and accelerate the degradation of PLGA in return, which is so-called autocatalysis [7–9]. In addition, the acidic environment may contribute to the denaturation of proteins or saccharides, finally influence the stability and bioactivity of drugs, therefore PLGA MS have been designed to combine with other polymer materials to improve their drug stabilities and release behaviors [10]. Chitosan (CS) is a positively charged, chitin de-acetyl derivative with great biomechanics, biocompatibility and biodegradability, and widely used for making MS to control drug release, as well as to protect it from being degraded [10–12]. In our previous research, we electrosprayed smaller PLGA MS and put them into CS solution to fabricate larger PLGA@CS composite MS by emulsification [13]. KSL-W, a kind of antimicrobial peptide, was previously dissolved in PLGA solution before electrospraying and in CS solutions before emulsification. It was found that outer peptides on the surface of composite MS got a burst release by diffusion, and then the inner peptides got a sustained release with the diffusion and microspheres’ degradation [14].

As we know, emulsification technique is a traditional and popular technique used for preparing MS with emulsifiers and surfactants [15–18]. However, it is a challenge to get highly uniform particle size by emulsification [19–21]. When particle size increases, it would take a longer time to degrade the MS and diffuse the loaded drugs and the degradation products from the center of microspheres. Therefore, different sizes of PLGA MS would experience different degradation and drug-release behaviors with inconsistent acidic stimulation from degradation by-products [22, 23]. Besides, the use of emulsifier and surfactant increased the risk of protein denaturation and impaired bioactivities, which should not be ignored. Electrospraying is another technique to fabricate MS in the Taylor cone-jet mode [14, 24–28], exerting an electric field on the emulsion solution to spray particles and collecting with an aluminum plate. The electrospraying MS turned out to possess consistent particle sizes, good drug encapsulation efficiency as well as user-friendly preparation process. But previously electrospraying was only used to make single MS while hasn’t been studied to make composite microspheres. Thus, we think hierarchically electrospraying might be an excellent substitution for traditional double-emulsification method to prepare composite microspheres.

In this study, we developed a hierarchically electrospraying process to prepare PLGA@CS composite microspheres, for purposes of simplified process, uniform particle sizes and controlled release behaviors, as well as better encapsulation efficiency (EE) and stability of loaded drugs.

## Materials and methods

### Materials

Chlorhexidine acetate (CHA) and bovine serum albumin (BSA) were obtained from Aladdin Chemistry Co, Ltd. (Shanghai, China). PLGA (75:25, Mw ≈ 50 kDa) was purchased from Medical Equipment Research Institute (Jinan, China). CS (Mw ≈ 100–300 kDa) was purchased from Bailingwei Science and Technology Co, Ltd. (Beijing, China). All other chemicals used were of analytical grade and obtained from Chemical Reagent Co, Ltd. (Beijing, China).

### Preparation of PLGA microspheres

PLGA MS were prepared by electrospraying. Briefly, 60 mg PLGA was dissolved in 1 ml trichloromethane. Different amounts of CHA and BSA (2.5 or 5 mg) were dissolved in 25 μl of deionized water and then added to the PLGA solution to form W/O emulsion by sonication on ice. The emulsion solutions then were electrosprayed under ambient atmosphere. Briefly, the emulsion solutions were placed in a 1-ml syringe and passed through a blunt stainless-steel nozzle at a constant flow rate of 1 ml/h using a micro-infusion pump. The applied voltage to the nozzle tip was 7 kV, and the distance between the nozzle tip and the aluminum collection plate was 20 cm. The organic solvent was removed by evaporation during the electrospray process, leaving solid MS on the collection plate. The PLGA MS were then lyophilized for 24 h to remove the residual solvent. PLGA MS of CHA-loaded, BSA-loaded and BSA+CHA-loaded were prepared, respectively.

### Preparation of CS microspheres

As stated above, 300 mg CS powder was dissolved in 10 ml 2% aqueous acetic acid solution. The solution was stirred magnetically for 2 h at room temperature, during which different amounts of BSA or CHA were added into the mixture solution. After that, mixture solution was poured into a 10-ml syringe and passed through a blunt stainless-steel nozzle at a constant flow rate of 5 ml/h using a micro-infusion pump, the receiving solution was 5% sodium tripolyphosphate (TPP) solvent, and the applied voltage to the nozzle tip was 10 kV.

### Preparation of PLGA@CS composite microspheres

**Figure 1:**
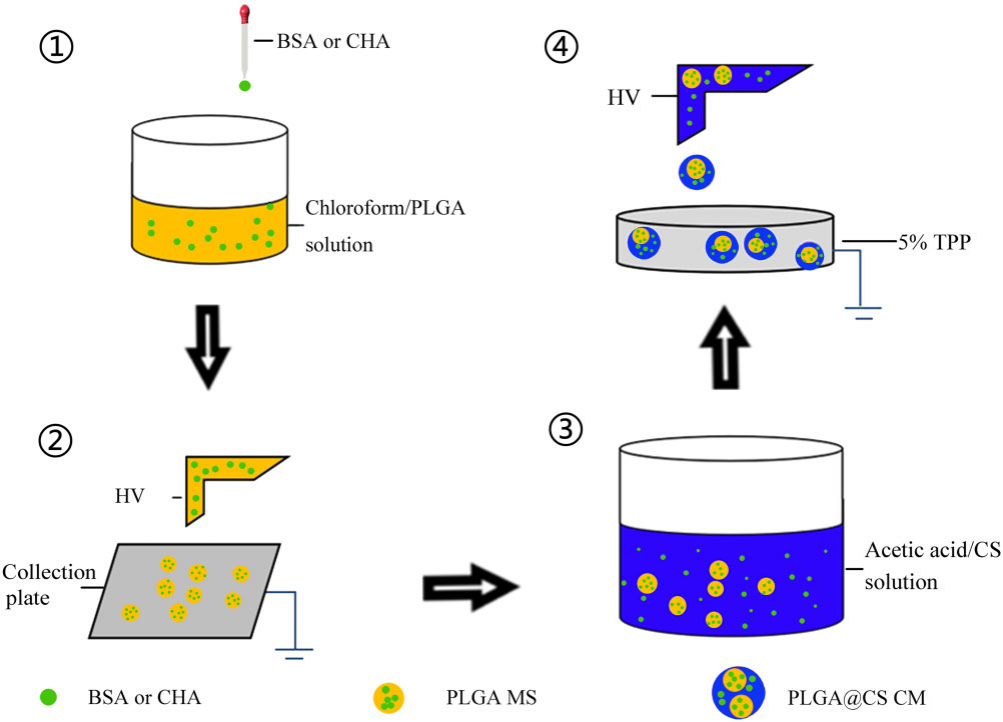
The schematic representation of hierarchically electrospraying process. PLGA MS were prepared by the electrospraying process, and then the PLGA microspheres, BSA and CHA were added into CS solution. Finally, the mixture was stirred magnetically to form uniform solution for electrospraying

**Figure 2:**
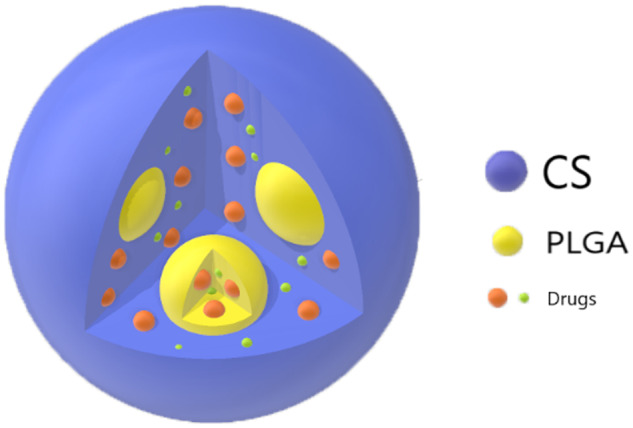
Patterns of CHA or BSA in PLGA microspheres, CS MS and PLGA@CS composite microspheres

CHA and BSA-loaded PLGA@CS composite MS were prepared by combining electrosprayed PLGA MS into CS MS (Figs 1 and 2). Firstly, 2% aqueous acetic acid solution was used as solvent to dissolve CS at a concentration of 3% (w/v), stirred magnetically for 2 h. Then, 60 mg of different formulations of PLGA MS were added to 10 ml CS solution, respectively, whilst stirred magnetically at room temperature for 3–4 h, during which different amounts of BSA and CHA (0, 2.5 mg) were also added to the PLGA/CS mixture. After that, the PLGA/CS/drug mixture was poured into a 10-ml syringe and passed through a blunt stainless-steel nozzle at a constant flow rate of 0.5 ml/h using a micro-infusion pump. The applied voltage to the nozzle tip was 10 kV, and the distance between the nozzle tip and the aluminum collection plate filled with 5% TPP solvent was 20 cm. The acetic acid solvent was removed during the electrospray process by evaporation, leaving solid MS on the collection solvent. The complex MS were then cross-linked with 5% TPP for 4–5 h. The final PLGA@CS composite MS were washed three times with deionized water to remove the residual cross-linking solvent. Finally, BSA and CHA-loaded PLGA@CS composite MS were lyophilized for 48 h at −25°C and 10 Pa. The prepared PLGA@CS composite MS were then preserved in −20°C refrigerator until use.

### Morphologic characterization of CHA and BSA-loaded microspheres

The shape, surface and particle diameter of CS microspheres, PLGA MS and PLGA@CS MS were observed by scanning electron microscopy (SEM) (LEO Gemini 1530 Field Emission Gun SEM, Germany). Samples were sputter-coated with platinum under vacuum and examined at the voltage of 10 kV. In order to confirm the distribution of PLGA in CS, the MS were dyed with fluorescent dyes, rhodamine (PLGA) and FITC (CS), and then observed by laser confocal microscope (Zeiss LSM710, excitation wavelength of 488 nm, Carl Zeiss Ltd, Oberkochen, Germany).

### Encapsulation efficiency of CHA and BSA-loaded microspheres

The EE of BSA and CHA in PLGA, PLGA@CS and CS MS were carried out as follows. For PLGA microspheres, a certain amount of PLGA MS were dissolved in dichloromethane, and the BSA and CHA were leached into distilled water. The concentration of BSA was measured with Coomassie brilliant blue G-250 (UV absorbance at 595 nm). CHA concentration was analyzed with ultraviolet spectrophotometry at 265 nm (Molecular Devices, Sunnyvale, CA).

For PLGA@CS composite microspheres, the BSA and CHA contents were determined as follows: 20 mg of composite MS were dissolved in 2 ml of 3% aqueous acetic acid solution for ∼15 min. After composite MS were dissolved, the supernatants were segregated by centrifugation. The precipitate was then dissolved after adding 0.7 ml acetonitrile and 1.3 ml, 0.01 M hydrochloric acid solution while stirring. After centrifugation, the supernatants were collected again. The first and second supernatants were combined and measured for CHA and BSA concentration.

For CS microspheres, the EE were determined as follows: certain amount of CS MS were dissolved in 4 ml of 3% aqueous acetic acid solution for ∼15 min, after MS dissolution, the supernatants were collected for testing CHA or BSA concentration.

$$\mathrm{Encapsulation}\;\;\mathrm{efficiency}\;\;\%=\left(\frac{\mathrm{Amount}\;\;of\;\;\mathrm{BSA}\;\;\mathrm{and}\;\;\mathrm{CHA}\;\;in\;\;\mathrm{microspheres}}{\mathrm{Amount}\;\;of\;\;\mathrm{BSA}\;\;\mathrm{and}\;\;\mathrm{CHA}\;\;\mathrm{added}\;\;\mathrm{initially}}\right)\times 100$$

### *In vitro* drug release profiles

*In vitro* release behaviors of BSA and CHA from MS were evaluated as follows. Briefly, 20 mg PLGA, CS or PLGA@CS MS were introduced into centrifuge tubes containing 2 ml PBS solution. Then, samples were incubated at 37°C in a shaking bath (Model THZ-C, Taicang Laboratorial Equipment Factory in China) at 60 rpm. At pre-determined time intervals, samples were centrifuged at 6000 rpm for 5 min. About 1 ml of supernatant was retrieved and replaced with 1 ml of fresh PBS solution. The concentrations of BSA were measured with Coomassie brilliant blue G-250 (UV absorbance at 595 nm), CHA concentration was analyzed with ultraviolet spectrophotometry at 265 nm. Three repeats for each sample group were conducted.

### Stability assessment of the BSA and CHA

The structure stability of the BSA and CHA after encapsulation was assessed by analyzing the secondary structure and molecular weight, just as described previously [14]. The secondary structure of the BSA released from MS was measured by Far-UV circular dichroism (CD, J-715-150L, JASCO, Tokyo, Japan). Briefly, the released drug solution was put into a quartz cuvette at room temperature. Then the path length, scanning speed and wavelength were set to 0.1 cm, 100 nm/min and 190–260 nm, respectively. Matrix-assisted laser desorption/ionization time-of-flight mass spectrometry (MALDI-TOF-MS, Autoflext, Bruker Daltonic Inc., Bremen, Germany) was also used to measure the molecular weights of the BSA and CHA released from composite MS as follows. Briefly, 2 μl of 4-hydroxy-cinnamic acid was mixed with 2 μl of released solution uniformly and the mixture solution was dried at room temperature to 1 μl. Then, samples were further dissociated by a nitrogen gas laser under the cationic reflector mode. Lastly, the peak signals (*m*/*z*) of the samples were collected and analyzed.

### Statistical analysis

The data were expressed as the means ± standard deviation (SD), *n* = 3. PRISM (GraphPad Software) was used to conduct a statistical analysis, using one-way ANOVA. Differences were considered statistically significant when the *P* < 0.05.

## Results

### Morphologic and structural characterizations of drug-loaded microspheres

**Figure 3:**
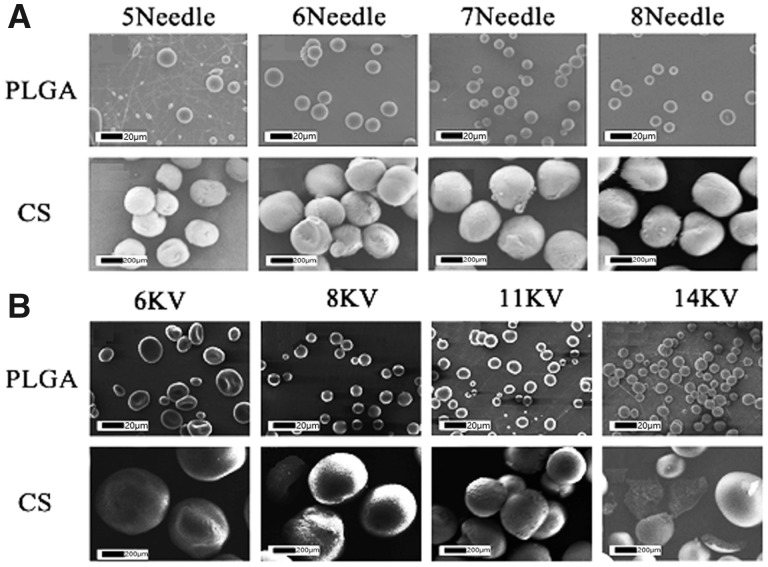
SEM images of PLGA or CS single-electrospray MS with different needle sizes (**A**) and voltages (**B**)

The SEM images of single-electrosprayed MS were shown in Fig. 3. It can be seen that all the MS were spherical and dispersed well with uniform particle size and smooth surfaces. For analyzing the influence of voltage and injection needle diameter on the particle size of the electrosprayed microspheres, we used voltages ranging from 6 to 14 V and syringes from 5th to 8th (∼0.25–0.58 mm in inner diameter). The results showed that, as the diameter of the syringe needles changed from 6th to 8th, the morphology of the PLGA MS evolved very slightly (Fig. 3A). But when we used 5th needle, the PLGA MS lost the ideal round morphology. Moreover, when we adjusted the voltage from 6 to 14 kV, the particle sizes of PLGA MS had a downtrend (Fig. 3B). However, we found that the PLGA MS became spheroid at 6 kV and filamentary at 14 kV, unlike spheres at 8 and 11 kV. Similarly, accompanied with the needle diameter decreasing, the particle size of CS MS had a declining trend (Fig. 3A). The CS MS became smaller when voltage was turned up from 6 kV, and finally fractured at 14 kV (Fig. 3B). By adjusting the needle diameter and voltage, we got the PLGA MS with 7∼15 µm in diameter and CS MS with 200–500 µm in diameter.

**Figure 4:**
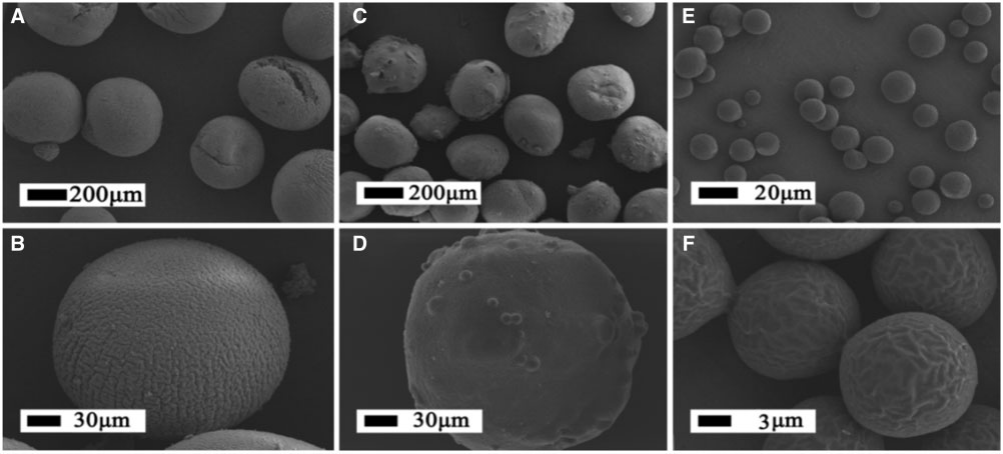
SEM Images of BSA and CHA-loaded CS MS (**A**, **B**); BSA and CHA-loaded PLGA@CS MS (**C**, **D**); BSA and CHA-loaded PLGA MS (**E**, **F**)

Based on the above-mentioned result, we adopted the parameters of 8 kV and seventh of the voltage and injection needle diameter for preparing drug-loaded PLGA, CS and PLGA@CS microspheres. As can be seen in Fig. 4, the electrosprayed drug-loaded PLGA MS exhibited crumpled surface and quite uniform particle diameters around 8–10 µm. Correspondingly, composite MS and CS MS prepared with 3% CS revealed spherical shapes, rough surface and quite uniform particle diameter around 250–300 µm.

**Figure 5:**
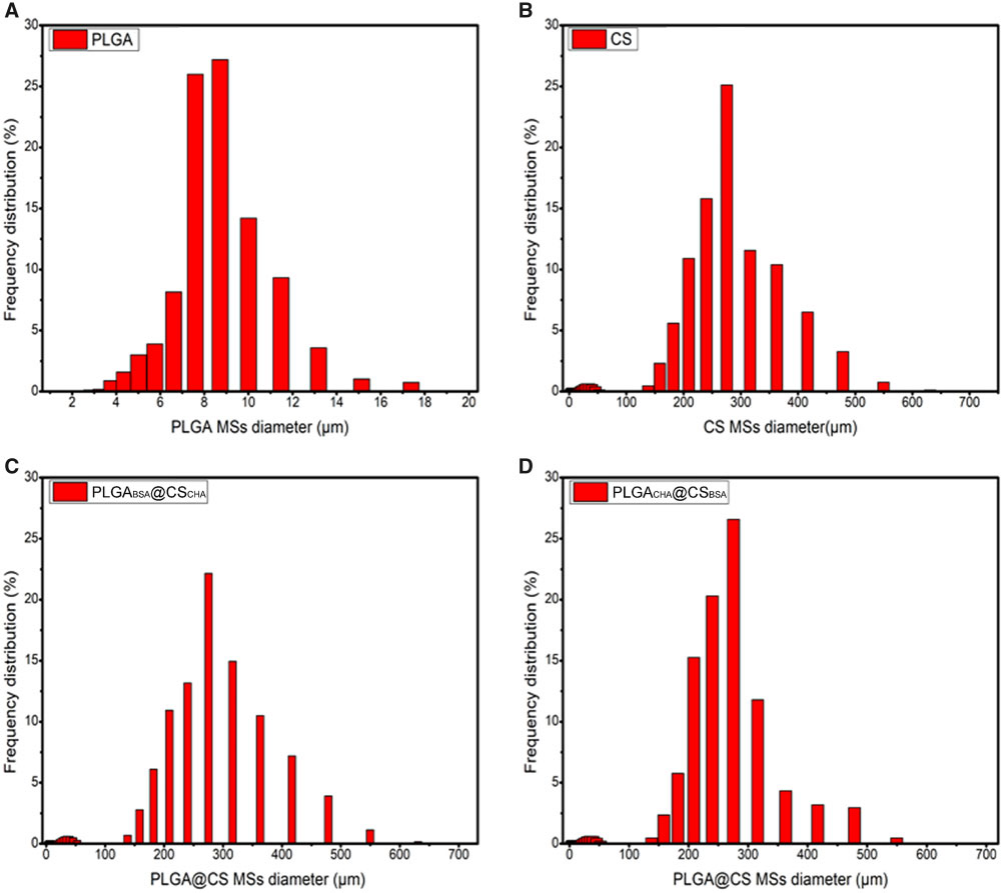
Size distributions of (**A**) PLGA, (**B**) CS and (**C**, **D**) PLGA@CS MS

The particle sizes of PLGA, CS and PLGA@CS MS were evaluated by laser particle size analyzer (Mastersizer 2000, UK). As can be seen in Fig. 5, the particle sizes of PLGA MS were mainly 8–10 µm and that of CS and PLGA@CS MS were 250–450 µm.

### Fluorescence stains of PLGA@CS microspheres

**Figure 6:**
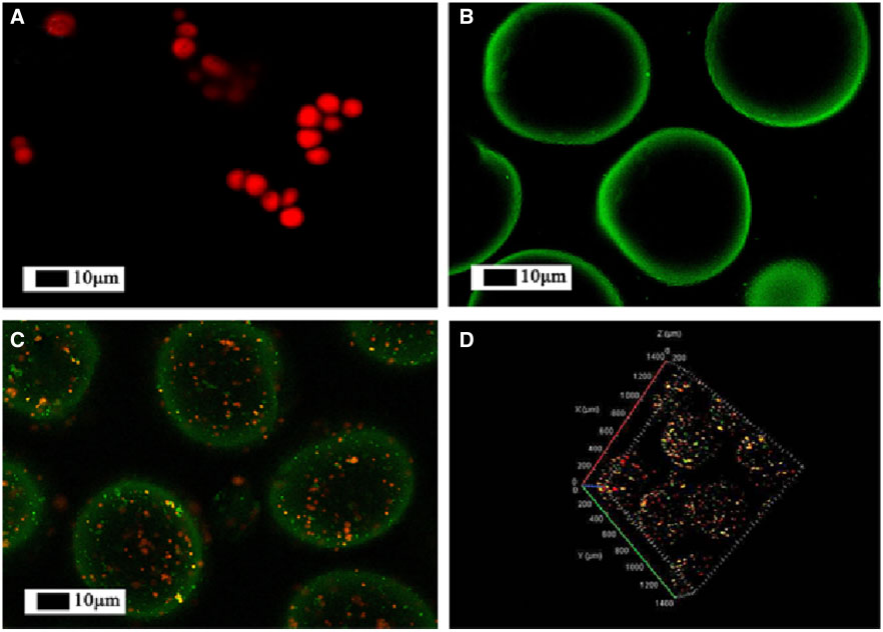
Fluorescence images for microspheres. Rhodamine B (red) and FITC (green) were chosen as the model drugs' labels. PLGA MS (**A**), CS MS (**B**), PLGA@CS MS (**C**, **D**)

In order to confirm whether the PLGA MS were successfully wrapped inside the CS microspheres, PLGA and CS were dyed with rhodamine B and FITC, respectively, which showed red or green under the laser scanning confocal microscope. The cross-section sweep and 3D transmission were observed. Figure 6A showed the rhodamine B-labeled PLGA microspheres, and Fig. 6B exhibited the FITC-labeled CS microspheres. As for the PLGA@CS composite microspheres, it can be clearly seen that PLGA was uniformly distributed in the CS MS to form a sphere-in-sphere composite structure (Fig. 6C).

### The encapsulation efficiency of PLGA, CS and PLGA@CS microspheres

The encapsulation behaviors of model drugs in the MS were characterized as the EE, as listed in Table 1. The PLGA MS fabricated by electrospraying had extremely high EE of over 90%. In CS microspheres, it was around 90% and that in the PLGA@CS MS could reach 90% as well. There were no significant differences between the EEs among the different drug-loaded composite microspheres. That is to say, we could control the amount of drug encapsulated in MS easily and accurately.

**Table 1:** The mean diameter and EE of the PLGA, CS and PLGA@CS microspheres

	MS formulations	BSA and CHA (mg)			Mean diameter (μm)	EE (%)
		BSA	CHA	Total		
PLGA MS	PLGA_BSA_	5	0	5	7.1 ± 0.6	BSA/92.2 ± 3.6
	PLGA_CHA_	0	5	5	7.0 ± 0.4	CHA/90.8 ± 2.6
	PLGA_BSA+CHA_	2.5	2.5	5	7.0 ± 0.3	BSA/92.1 ± 3.2
						CHA/91.6 ± 2.6
CS MS	CS_CHA_	0	5	5	260.3 ± 23.3	CHA/88.5 ± 5.3
	CS_BSA_	5	0	5	266.2 ± 19.3	BSA/90.5 ± 4.5
	CS_BSA+CHA_	2.5	2.5	5	264.3 ± 26.3	BSA/89.7 ± 3.8
						CHA/90.6 ± 4.3
PLGA@CS MS	PLGA_CHA_@CS_BSA_	2.5	2.5	5	290.4 ± 24.3	CHA/93.5 ± 3.7
						BSA/89.3 ± 4.6
	PLGA_BSA_@CS_CHA_	2.5	2.5	5	288.2 ± 17.4	BSA/93.1 ± 3.6
						CHA/92.3 ± 4.6
	PLGA_BSA+CHA_@CS	2.5	2.5	5	294.4 ± 12.3	BSA/92.4 ± 1.6
						CHA/91.6 ± 2.7
	PLGA_CHA+BSA_@CS_CHA+BSA_	2.5	2.5	5	289.6 ± 20.8	BSA/90.9 ± 3.7
						CHA/89.9 ± 5.7

### *In vitro* release profiles

**Figure 7:**
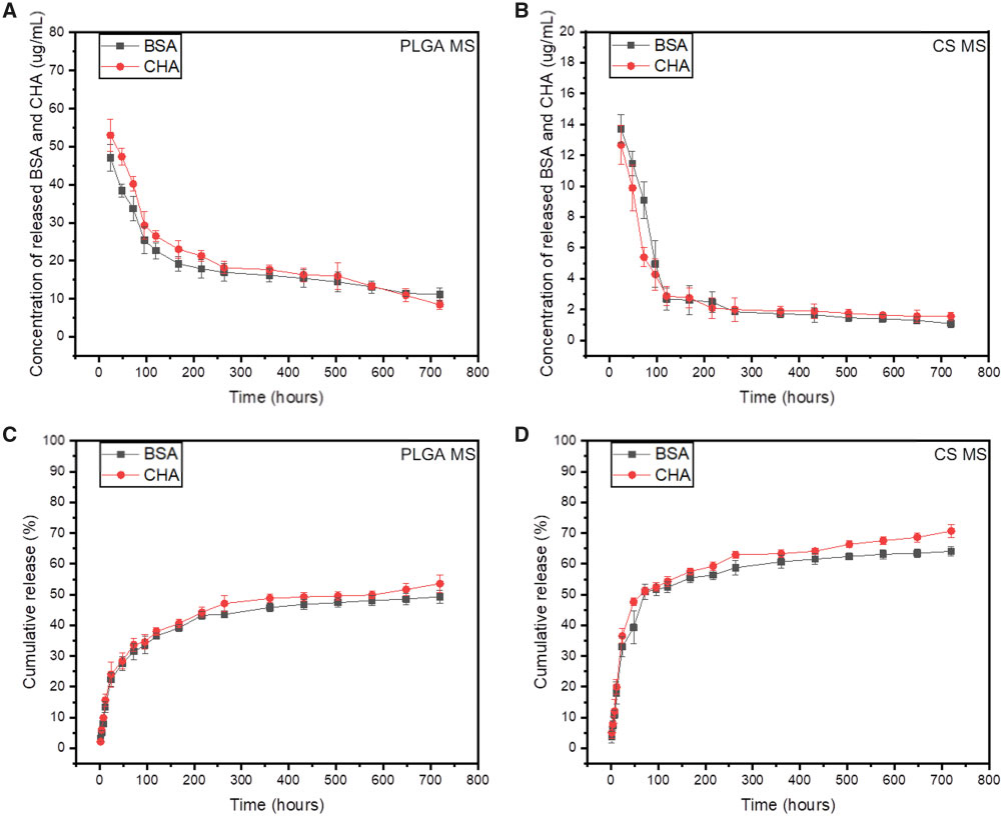
The concentration profiles and cumulative release profiles of released BSA and CHA in PLGA MS (**A**, **C**) and CS MS (**B**, **D**). Data are shown as the means, *n* = 3

**Figure 8:**
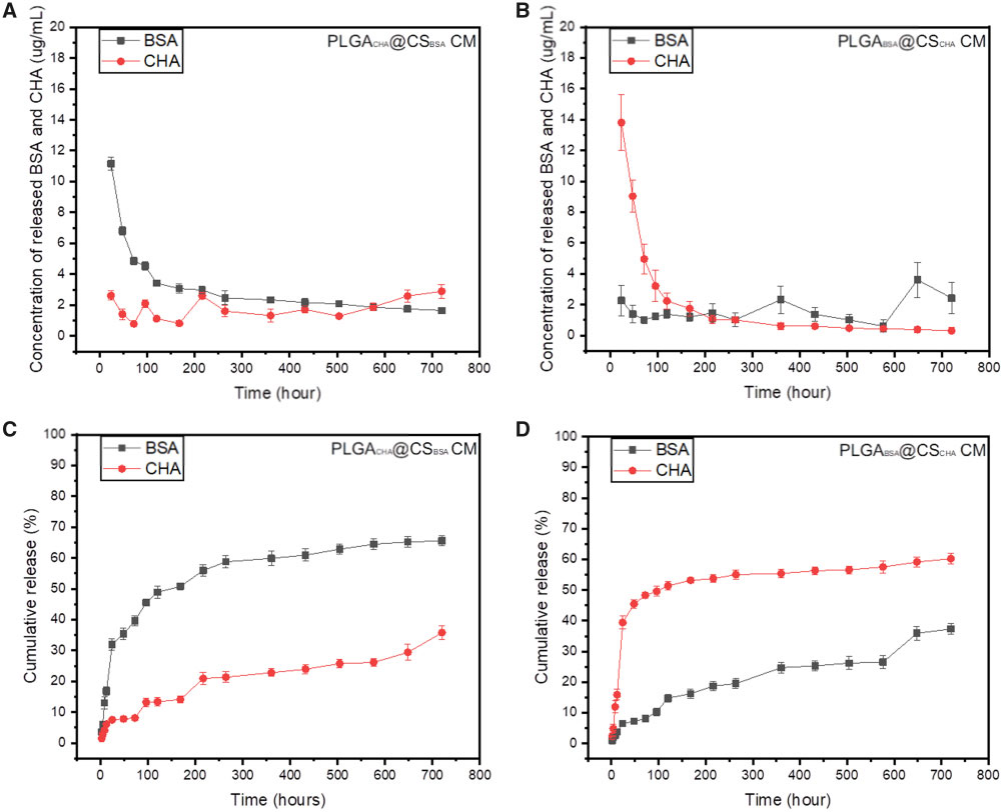
The concentration profiles and cumulative release profiles of BSA and CHA from PLGA_CHA_@CS_BSA_ (**A**, **C**) and PLGA_BSA_@CS_CHA_ (**B**, **D**) composite microspheres

In order to explore the drug release property, we selected BSA and CHA as model drugs to load and analyzed the release behaviors in MS of different formulations. BSA and CHA are proteins with greatly different relative molecular mass, so we could eliminate the selection bias of molecular weights. The quantities of BSA and CHA were detected by Coomassie brilliant blue G-250 (UV absorbance at 595 nm) and ultraviolet spectrophotometry (UV absorbance at 265 nm), respectively. Figure 7 showed the concentration profiles and cumulative release profiles of the released BSA and CHA from single MS PLGA MS or CS MS, whereas Fig. 8 showed the profiles from PLGA@CS composite microspheres. Generally speaking, the release profiles of both CHA and BSA from PLGA microsphere were time dependent, and turned out to be an initial rapid burst release followed by a relatively slow and sustained release (Fig. 7A). As for dual-drug-loaded PLGA microspheres, ∼37.2 ± 2.6% of BSA and CHA got released from PLGA MS in first 3 days (Fig. 7C). Then, over 52.8 ± 1.3% of the total BSA and CHA were released within 30 days. For the dual-drug-loaded CS microspheres, the release rates of CHA and BSA were similar to that in PLGA MS during the entire release period (Fig. 7B). Within 3 days, the accumulative release amount of BSA and CHA released from CS MS was 50.2 ± 1.8%, and after 30 days of incubation, 70.3 ± 2.4% of BSA and CHA were released (Fig. 7D).

In regard to the composite microspheres, we speculated the outer CS shell can prevent the buffer solution from infiltrating into the inner PLGA MS at the initial stage. When the CS shell gradually decomposed, the inner PLGA MS began to expose and then degrade gradually. To confirm it, we fabricated sets of PLGA@CS composite MS loaded with different drugs. In one group, BSA was encapsulated in PLGA MS while CHA was in CS MS, and in the other group CHA was encapsulated in PLGA MS while BSA was in CS MS. It is interesting that the release rates of drugs from PLGA MS were much slower than CS shell (Fig. 8). It means when PLGA MS were embedded in CS microspheres, the release activities from PLGA MS can be further controlled.

### Stability assessment of loaded drugs

**Figure 9:**
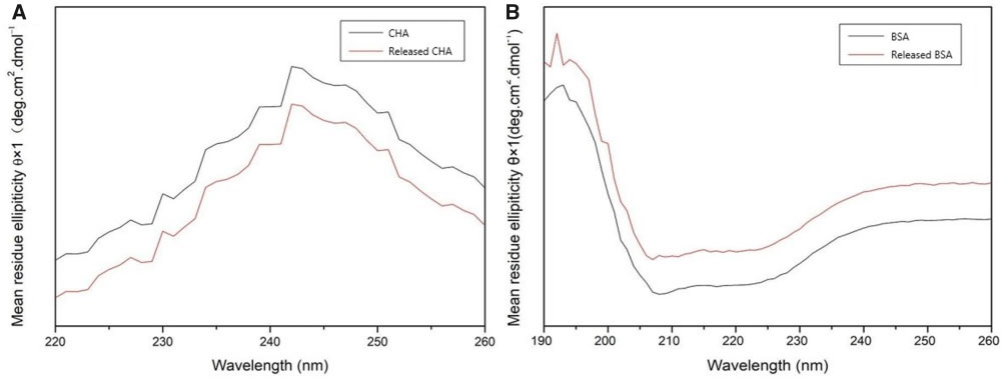
Far-UV CD results of CHA (A) and BSA (B) that were original and released from composite microspheres

To evaluate the stability of the released drugs, the secondary structure and relative molecular mass were analyzed. As seen in the curves of Far-UV CD, the positive peak at 243 nm and the curve of CHA from PLGA@CS composite MS matched the original CHA well (Fig. 9A). Similarly, the negative peak at 207 nm, the positive peak at 192 nm and the trend of BSA from composite MS were all consistent with theoretical value (Fig. 9B). It meant that after encapsulation, the secondary structures of drugs were still stable as compared with original drugs.

**Figure 10:**
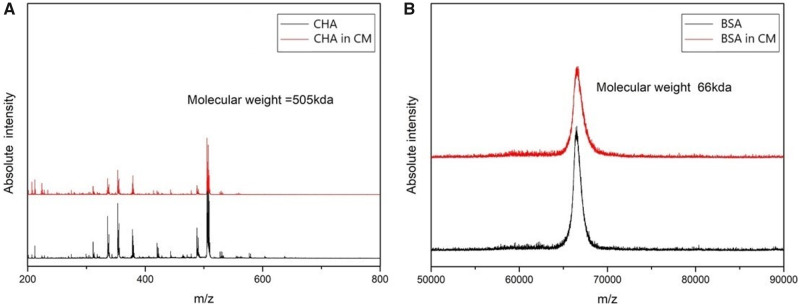
Mass spectrometry spectra of the CHA and BSA before (**A**) and after (**B**) encapsulations

The relative molecular weights of BSA and CHA were also obtained from the peaks of the spectrums (Fig. 10). Compared with original BSA, the relative molecular mass remained at 66 kDa. In the same way, the CHA from composite MS remained the normal relative molecular mass of 505 kDa. The analysis results were both consistent with their theoretical values. Therefore, we speculated that no obvious degradation happened to BSA and CHA throughout the procedures of encapsulation and storage, which is a key prerequisite for protein drugs to function accurately.

## Discussion

The MS have been developed as a sustained-release carrier for a long time, and the preparation techniques of the MS have also undergone various evolutions like emulsification to electrospray [29]. Emulsification is a traditional and mature method, but the MS prepared have certain defects such as inconsistent particle sizes and compromised drug-loading rates, which limit related applications. Furthermore, the complicated procedures including the long and complicated stimulations of multiple reagents to loaded drugs, as well as the safety risk for operators, should be taken seriously. The problems remain when preparing composite MS for multi-stage drug loading.

A practicable solution is to utilize electrospray technique to fabricate microspheres. This technique proved practical to uniformly and accurately control particle sizes with several to several hundred micrometers in diameter, and needed very few chemical reagents. Therefore, we adopt a hierarchically electrospraying technique instead of the traditional double-emulsification to prepare composite microspheres. In our experiment, we successfully fabricated composite MS by hierarchically electrospraying PLGA and CS, and adjusted the voltage and needle diameters for different particle sizes. The diameters of the composite MS were quite uniform, mainly between 200 and 300 μm, which were a bit bigger than the PLGA/CS composite MS reported previously [30]. But our MS remained very small and could be encapsulated in multiple medical scaffolds. In order to encapsulate enough PLGA MS inside the CS microspheres, the PLGA MS were adjusted to be 5–10 μm in diameter. Besides, each kind of composite MS prepared revealed a high EE of more than 90%. Rhodamine B and FITC staining were used to identify the composite structure of PLGA@CS composite microspheres, and we could find the small PLGA MS were uniformly dispersed in large CS microspheres.

The composite PLGA@CS MS formulated core-shell structures, so we can put one kind of drug in inner PLGA cores and another kind of drug in outer CS shells. Considering the release profiles and the potential applications of the MS for tissue engineering, we evaluated the release profile over 30 days. Then, we found that the release of the drugs from the MS tended to be stable after 200–300 h with the burst release within the first 100 h, and the cumulative release percentage was about 50–60% after 300 h, regardless of the relative molecular weights. It meant we might get various combinations of multiple drugs in composite microspheres. If we need some drug to function early, we could put it in CS shells. And if we need some drug to function later, we could put it in PLGA cores. Similarly, if we put drug in both the cores and shells, it would function throughout the whole degradation process. We think the release profiles of the encapsulated drugs from the MS could also be adjusted by changing many parameters throughout the microsphere fabrication, including the properties of the materials and their degradation behaviors, sizes of the microspheres, the primary dosages of encapsulated drugs, distributions of the drugs inside the microspheres, even the physical and chemical properties of the drugs.

Compared with the MS prepared by conventional technique, the hierarchically electrospraying technique used in this experiment had various advantages. First, the EE of MS prepared by hierarchical electrospray can easily reach 90%, which was significantly more than emulsification’s 80% [31]. In addition, the MS prepared by electrospray revealed quite uniform particle sizes, which was too hard to be accurately controlled by traditional emulsification technique [13]. And most importantly, hierarchically electrospraying is a method that much more user-friendly than double-emulsification and effectively reduces the use of various chemical reagents. In a word, by hierarchically electrospraying, we can control the particle size, drug loading and safety of composite MS more accurately and easily than traditional emulsification.

Nevertheless, there may also be some potential disadvantages of existing hierarchically electrospraying. Taking an example, the collection efficiency of electrospraying, as well as hierarchically electrospraying, is relatively lower than double-emulsification. At the same time, we couldn’t completely avoid the use of organic reagents, for PLGA and CS need to be dissolved before electrospraying. Moreover, the electric filed exerted on the drug solution may get the drug charged, then the influences on the property and bioactivity of drugs remain to be evaluated [32]. Therefore, further researches are necessary to increase the accuracy of release control, simplicity as well as safety and therapeutic significance when we optimize the technique and deal with certain drugs before clinical applications. Furthermore, we are conducting a new study that mixing different PLGA MS loading different drugs, and then getting the mixture in larger CS microspheres. In this way, we are looking forward to realizing complicated sustained release of multi-drugs (≥3 kinds totally), so that we might formulate multi-function bioactive scaffolds to meet specific clinical requirements.

## Conclusion

In this study, we successfully developed a modified technique of hierarchically electrospraying to prepare PLGA@CS composite microspheres, which could load multiple drugs for multi-stage drug release in clinical treatment. The smaller electrosprayed PLGA MS were successfully packaged and uniformly dispersed into the larger CS MS by secondary electrospray. The composite MS not only possessed even morphology, but also exhibited multi-stage release behaviors. They had consistent particle sizes and round spherical shapes, and after freeze drying they remained a typical sphere with corrugated surface. By analyzing the loading rates of model drugs, we found the composite MS of hierarchically electrospraying could carry more drugs when compared with traditional double emulsification. To sum up, the hierarchically electrospraying technique reveals obvious improvements in both experimental process and results, which not only improves drug-loading rate but also saves manpower and material resources.

## Funding

This research was supported by National Natural Science Foundation of China (nos. 31771056, 81671827 and 51572144). 

*Conflict of interest statement*. None declared. 
