# Corrigendum to: Hierarchically electrospraying a PLGA@chitosan sphere-in-sphere composite microsphere for multi-drug-controlled release

**DOI:** 10.1093/rb/rbaa023

**Published:** 2020-08-08

**Authors:** Zhu Liu, Weilong Ye, Jingchuan Zheng, Qindong Wang, Guowu Ma, Huiying Liu, Xiumei Wang

**Affiliations:** r1 State Key Laboratory of New Ceramic & Fine Processing, School of Materials Science and Engineering, Tsinghua University, No. 1 Qinghuayuan, Beijing 100084, China; r2 Department of Prosthodontics, School of Stomatology, Dalian Medical University, No.9 west section, Lvshunnan Road, Dalian 116044, China

doi: 10.1093/rb/rbaa009, *Regenerative Biomaterials* 2020;381–390

The co-author, Guowu Ma who is the supervisor of the authors Zhu Liu, Weilong Ye, and Qingdong Wang, provided professional guidance on the experimental design and financial support for the experiments. Therefore, we’d like to declare a correction to the authorship that Prof. Ma is listed as one of the corresponding authors.

